# Qualitative exploration of service users and social prescribing link workers of the Armed Forces Community social prescribing scheme in Cornwall

**DOI:** 10.1136/bmjoq-2025-003842

**Published:** 2026-02-03

**Authors:** Marie J Polley, Michelle Tytherleigh, Helen E Seers, Chris Kent, Richard A Sharpe

**Affiliations:** 1 Research and Development, Meaningful Measures Ltd, Bristol, UK; 2 Institute for Connected Communities, University of East London, London, UK; 3 Division of Psychology, University of Chester, Chester, UK; 4 Active Plus, Health & Wellbeing Innovation Centre, Treliske, Truro, Cornwall, UK; 5 Public Health, Cornwall Council, Truro, UK; 6 European Centre for Environment and Human Health, University of Exeter, Faculty of Health and Life Sciences, Exeter, UK; 7 Health Determinants Research Collaborative (HDRC), Public Health, Cornwall Council, Truro, UK

**Keywords:** Mental health, Qualitative research, Veterans, Social Isolation, Social Prescribing

## Abstract

**Introduction:**

The Armed Forces Community (AFC) experience significant health inequalities and barriers to accessing support. Cornwall, England, has one of the highest AFC populations. A social prescribing service, delivered by Active Plus, was developed to improve physical and mental well-being and target wider determinants of health of this population. Uniquely, the service is delivered by social prescribing link workers who are themselves veterans.

**Objective:**

This evaluation aimed to qualitatively explore the experiences of the service users and the social prescribing link workers.

**Method:**

Semistructured interviews of five service users and four social prescribing link workers were conducted online using Google Meet. Service users were sampled to reflect diversity in service branch, age and time since leaving the Armed Forces. Informed consent processes were carried out. Data were transcribed, checked, anonymised and inductively thematically analysed.

**Results:**

Service users had struggled to transition from a military to a civilian identity. Referrals were for mental health, social isolation, housing, finances, physical health and domestic abuse. Having a social prescribing link worker who was a veteran was a crucial component of establishing trust, without the need to explain military culture or experiences. This was instrumental in helping service users engage more fully with the service offered. Service users reported improvements in their living conditions and mental health, were more connected to other people and had a renewed sense of hope and optimism.

**Conclusion:**

This is the first report of experiences of the AFC receiving bespoke social prescribing support. The findings highlight the crucial nature of designing the social prescribing service to resonate with military culture and the difficulties of transitioning to a civilian identity. Service users had improved well-being and were supported with a range of determinants of health. Further research needs to be carried out on other AFC members to confirm the findings.

**WHAT IS ALREADY KNOWN ON THIS TOPIC**

- It is known that the Armed Forces Communities (AFC) experience reduced service equity and health inequalities associated with a range of physical, mental and social health needs when leaving active service.

**WHAT THIS STUDY ADDS**

- These findings detail how and why the AFC benefit from a tailor-made social prescribing service for this population, especially ensuring that the social prescribing link worker is also from the AFC and understands military identity and culture.

**HOW THIS STUDY MIGHT AFFECT RESEARCH, PRACTICE OR POLICY**

- The findings offer insights into how best to serve the AFC when providing a social prescribing service and support the transition from military to civilian identity.

## Introduction

### Background

 The Armed Forces Community (AFC) experience reduced service equity and health inequalities associated with a range of physical, mental and social health needs.^1 2^ AFC individuals with poor mental health are at increased risk of suicidal ideation, especially among those who do not seek support for their mental health and well-being.^3 4^ Some researchers link the mental health issues to the idea of ‘warring identities’ where veterans are trapped between their Armed Forces identity and their new civilian identity after service.^5^ This transition from military to civilian life is often challenging and can be viewed as a change in one’s sense of self.^6^,^8^ For example, the risk of mental health and suicide for AFCs is influenced by a range of barriers to seeking support, as well as deployment experiences, stigma, military culture and a lack of understanding by civilian staff.^9^ Other risk factors include age, sex/gender, social isolation, adverse life events prior to joining, and drug and alcohol misuse.^10^,^13^ Poor mental health and comorbidities, such as drug and alcohol use, also often mean that individuals reach crisis point before they seek help.^14^ Veterans’ health and well-being cannot be solved solely by addressing one type of barrier alone.^15^ In the UK, there is an expectation that primary healthcare will record and support the AFC population through prioritised and specific services.^9^ Those working in healthcare, however, may not have the knowledge or understanding of the unique experiences of AFC members and of military and non-military services. This can further compound the barriers AFC face when seeking support.

In England, the National Health Service set out plans to increase access to social prescribing^16 17^ which helps link people to appropriate support for their health and well-being. In the general population, social prescribing has been found to improve the health and well-being of those who access this service in the primary healthcare and voluntary sector.^18^,^26^ There is still a need, however, to overcome inequalities in referrals to support minority groups in the population and those who are underserved.^27 28^

### Development of social prescribing for the AFC in Cornwall

**Figure 1:**
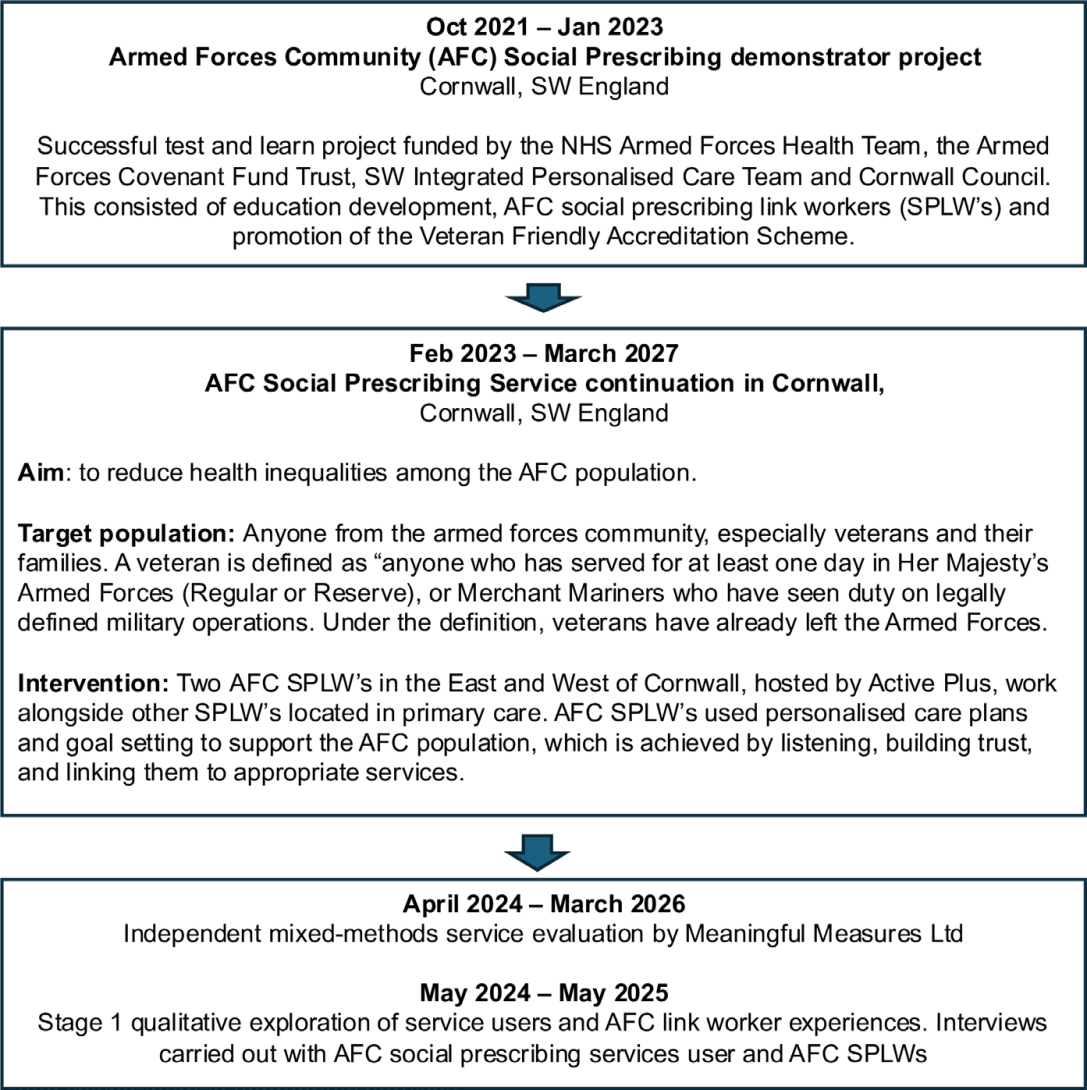
Development schedule of the Armed Forces Community social prescribing scheme and evaluation.

Cornwall, South West of England, has one of the highest AFC populations (6.3% of residents) when compared with England (3.8%).^29^ Given that the AFC is a minority group and arguably underserved, Public Health Cornwall Council funded an AFC Social Prescribing Service (AFCSPS) as part of its work to reduce health inequalities. This was based on an original AFC Social Prescribing Link Worker pilot (AFC SPLW), which improved mental well-being outcomes of those supported,^30^ consistent with social prescribing programmes.^31^,^34^ In August 2023, Public Health continued to fund AFC SPLWs for a further 3-year period. The AFCSPS is now delivered across Cornwall. This includes targeting the wider determinants of health such as social isolation, finances and employment (figure 1) and could be described as a holistic service according to Kimberlee.^35^ Referrals into the AFCSPS come not only from General Practitioners (GPs) and SPLWs in primary care but also from secondary care, community mental health teams, local Voluntary, Community, and Social Enterprises (VCSE) organisations, housing associations, local naval base, self-referrals and other community-based professionals.

The AFCSPS is currently delivered by two SPLWs who have lived experience of working in the Armed Forces and knowledge of specific AFC support. Most service users are met at community locations of their choice or at the Active Plus Health and Wellbeing Innovation Centre in Truro. Home visits (~5%) are carried out only when necessary. The SPLWs work with individuals to develop a personalised action plan and link AFC individuals to appropriate activities in the community, wider health and social care system and specific AFC support. Further detail on service provision can be found in online supplemental figure 1. The differences between the AFCSPS and a civilian social prescribing services are explained in online supplemental table 1.

Timely therapeutic and social prescribing interventions for AFC have been found to reduce depression and anxiety^36^ and prior research has explored the perspectives of SPLWs^37^ but has not fully explored the role of AFC SPLWs. As part of the 3-year funding, a two-stage evaluation was commissioned to better understand the benefits of social prescribing tailored for the AFC. Stage one, as reported here, documents for the first time the experiences of social prescribing for the AFC from both AFC SPLWs and AFC service users’ perspectives.

## Methods

### Design

To evaluate the AFCSPS, a qualitative exploration of staff and service users’ experiences was carried out.

### Procedure

The SPLWs invited service users who were not in crisis to participate in an online interview about their experiences of the service with independently commissioned researchers (MT and MJP). Service users were purposively sampled to achieve a range of genders, ages and Armed Forces served in. Five AFC individuals agreed to be interviewed. Reasons for not taking part were due to illness or being too busy. Two previously employed and two currently employed SPLWs were also invited to participate in interviews.

All participants were recruited using informed consent procedures. All participants received information sheets prior to their interviews and signed their consent to take part. Semistructured interviews were carried out by MT and MJP using Google Meet (see online supplemental figure 2 for interview schedules). Two previously employed SPLWs and all AFC participants were interviewed between May 2024 and May 2025. One dyadic interview with the two currently employed SPLWs took place in April 2025. Each interview lasted no more than 40–50 min.

### Data analysis

Interviews were recorded and transcribed using Google Meet, transcriptions were checked for accuracy, anonymised by MT and MJP, and the recordings then deleted. The transcripts were analysed using Braun and Clarke’s six-step process.^38^ An inductive analysis approach was used to allow the themes to emerge organically and potentially reveal insights beyond the initial focus areas. Quirkos V.3.0 software was used to code the data, and codes were shared and checked by MT and MJP for inter-rater reliability.

## Results

Overall, 293 people were accepted into the AFCSPS, age range was 16–84 and modal age category was 55–64 years. 23% of service users were female, 75% were male and 2% preferred not to say. Service users were split between the Army (including reserves and the Territorial Army) 51%; Royal Navy (including reserves, Royal Marines) 26% and the Royal Air Force (including reserves) 12%. 11% of service users were related to a veteran with no military service themselves.

Four of the five AFC participants were men, which is representative of the proportion of the service users in this service. Their ages ranged from 42 to 79 years, and all had left the Armed Forces between 1975 and 2013. Reasons for referral, marital status and Armed Forces served in were mixed to provide a range of experiences (see table 1). Of the SPLWs, two were female and two were male. They all served in either the British Army or Royal Air Force and left their service positions between 2016 and 2024.

**Table 1:** Participant information for service users who were interviewed

Sex	Marital status/dependents	Armed Force served with	Reason for referral to AFCSPS
Male (AFC1)	Single, no dependents	Royal Navy	Housing, mental health, social isolation, financial issues, domestic and sexual abuse
Male (AFC2)	Single, no dependents	Royal Air Force	Mental health
Male (AFC3)	Married/living with partner, three dependents	British Army	Housing, mental health, advocacy with GP
Male (AFC4)	Single, one dependent	Royal Air Force	Social isolation
Female (AFC5)	Single, no dependents	Royal Air Force	Mental Health, social isolation, physical health, financial issues

AFC, Armed Forces Community; AFCSPS, AFC Social Prescribing Service; GP, General Practitioner.

### Reasons for using the service

The SPLWs described a range of reasons why people use the AFCSPS. Some participants had left the Armed Forces many years ago. With issues not addressed at that time, SPLWs noted the complexity and/or number of long-standing/legacy issues participants had, for example, being homosexual in the Armed Forces. One SPLW described seeing more of the AFC coming to the service with PTSD and needing support to deal with their mental health as well as the negative knock-on effects of unsupported PTSD, such as on their families, relationships, use of addictive substances and housing issues.

The biggest things that I’ve noticed generally are that it’s PTSD related. It’s not necessarily mental health [or] to do with anything else. It’s the stuff they’ve seen and done in the military. So, they’ve had to go 5, 10, 15, however many years of just struggling and coping…And it has a knock-on effect with their families. …. The wives have left them, they can’t see their children, which means they've turned to alcohol or drugs, and then that’s then lost their house, and they haven’t and it’s just a massive downward spiral (SP1)

All participants in this evaluation generally had a complex mixture of ongoing support needs, which impacted them in different ways. Several needed support with their housing and heating or were unable to keep on top of the house due to their ill health. For older participants, they did not understand how to do practical things which required a computer or internet access. Many participants explicitly or implicitly highlighted financial difficulties, for example, one participant had very little food to eat.

They could understand, right from the beginning, that I was totally strapped for cash. That I was lonely…They [SPLWs] began to understand that I was cold. I couldn’t afford …. In fact, my heating was broken…They began to understand all of those things. (AFC1)

I’ve been ill on and off for years. However, it’s gotten worse. And I was just mentioning to people that I have trouble doing things around the house…but I first got introduced to social prescribing … because I went to my doctor because I have ongoing health issues and they asked, ‘would you be interested in social prescribing?’ (AFC5)

Unsurprisingly, mental health issues were also mentioned for most participants. Many of the participants explained that they were socially isolated, and if that was not explicitly said, it was often picked up on by the AFC SPLWs.

Mental health and finances and social isolation often are very much linked in because if somebody’s feeling worried about their finances, it will affect quite likely their mental health and then that quite likely will mean that they're socially isolated. (SP2)

…through the chat you’re having and the rapport you’re building, you suddenly realise that they are socially isolated. (SP4)

### Supporting the transitioning from an Armed Forces identity to a civilian identity

Many dimensions of Armed Forces identity emerged in the analysis of the participants’ experiences of the AFCSPS. This is described in more detail below.

Unique to this veteran social prescribing service was the fact that the link workers were all veterans themselves and therefore could identify with the Armed Forces perspective of the participants. Both the AFC participants and AFC SPLWs highlighted how crucial receiving support was from an AFC SPLW who was a military veteran and genuinely understood and empathised with their military experience. Having this identity and understanding broke down communication barriers helped AFCs feel truly heard, enabled an authentic connection and helped build trust. The AFC identity is also related to language, so for SPLWs to be able to understand the ‘banter’ was an added benefit.

Straight away, it is somebody who kind of gets me and I don’t have to be a completely different person by the way I am interacting with them. (AFC5)

It is a level of understanding and empathy that cannot be achieved by a GP social prescribing link worker. They just don’t have it… I’m able to go in where they’re at because I’ve been there. (SP2),

Each AFC has to make their own unique transition to civilian life and find their new identity. The SPLWs were able to understand how difficult the transition can be. Coming from different service branches themselves, the SPLWs tried to tailor their support to this unique service experience rather than provide support based entirely on geographic location. The SPLWs were also able to understand how the sense of belonging to the service community endures, irrespective of how long ago a person left the Armed Forces. For example, one AFC described the Army as being their life. One SPLW also described how continuing to be part of the AF Community was their motivation for taking on that role:

We also are very mindful because each veteran is an individual with separate personal needs and if I’ve got a veteran in the East, for example, that might be in the RAF, I will go to [my colleague] and say can you give me your experience, give me your views so we work together to give the best possible support to that veteran regardless of where they live (SP2)

…a lot of the veterans that you tend to work with have clung on to that identity of being a veteran and being in service… It’s like, you know, they they may have left the military or the service 20 years ago, but that’s what they’ve held on to, you know. …They haven’t progressed from there … They believe that’s it. (SP4)

By drawing on their own Armed Forces identity and adopting a military mindset approach to support and structure, both SPLWs and the AFCs they support were able to work within a reliable and familiar (military) way to find solutions. One SPLW also described how AFC would often welcome a blunter and straight-talking approach, and how this differed in civilian life; another benefit of having an SPLW who is also an AFC.

A focus on ‘getting the job done’ and ‘not letting things fail’ and supporting each other to work as a team were all hallmarks of military operations and very much linked to the Armed Forces identity. Building on the work started by the two original SPLWs, the current SPLWs operated the service as a standardised system. This included using a very structured, efficient and results-oriented approach, and a pragmatic approach to problem-solving:

The military mindset, it’s very much that we won’t let things fail… the mindset is, you know, we have to get to that result. (SP3).

As long as the focus is on the support, the welfare, the safety of our veterans, we’ll just do what needs to be done, whatever that is. (SP2)

Being in the AFC, as well as a SPLW, the SPLWs have tried to manage their own military mindset linked to their own aspects of Armed Forces identity, by setting expectations with their clients and maintaining professional boundaries.

So, we manage that by sort of regular check-ins with each other and discussing about the support that we’re giving to people because we’re both aware that we both have a tendency to over-support. That can be something that we have to be really aware of because it’s not always the best thing for them. It’s knowing when to step back. We both got a lot better at it as we’ve evolved in our roles. Initially I think that we both probably struggled with that. (SP2)

The AFC are used to structure, and for those struggling with a complexity of issues, the need for consistency and a structured ‘one issue at a time’ approach was also found to make things more manageable and less overwhelming. AFCs also appreciated the amount of effort the SPs put in often to go ‘above and beyond’:

It’d be best if everyone just knocks me off their to-do list so I can get one person, one organisation to say, right; we can do this and, like, you know, and have it done. Yeah, I get so confused. I’ll get bombarded with different organisations that, in the end, I just bury my head in the sand because I can’t take it. It’s too much information. Yeah, and I don’t know. … don’t understand half of it (AFC4)

…she’s very good at keeping me to the point and militarised…. So, if I try to wander off track, no ….We’re dealing with this right now…She would always try to get the most priority one and once she started on it, she would finish that project. Then we move on to the next one. (AFC3).

### The benefits of the service

The AFC in this evaluation particularly reported having support to chase up medical appointments for their mental health and help with practical issues in the home or practical issues that needed to be done online. For some, the SPLWs were supporting the most basic of human needs such as access to food and heating.

And the joy it brought when the Food Bank brought me a lovely parcel of food. I was beside myself with happiness… (AFC1)

About one of the first things they did for me was to make a referral and talk to [xxxx] at the Community Energy Plus. (AFC1)

[SPLW] gets things that I do need, but I don’t know how to get them myself. I haven’t got a computer in the house or anything like that so it’s all done by my phone. And I wouldn’t have been able to have the access to what [SPLW] can get hold of (AFC4)

Social isolation was something several of the service users were referred to the AFCSPS for and SPLWs were able to find the groups and communities to support the AFC participants to become part of.

It’s got me out in the house, and if, if it wasn’t for people like yourselves…I’ll just be stuck in doors. (AFC4)

…then the social prescribing … got me out doing more crafts because I am into my craft works and things like that. (AFC5)

The participants often described the impact of the SPLW support as making them feel more optimistic and hopeful for the future. For some, the AFCSPS had provided a crucial turning point for them, moving them from a state of despair to a more positive outlook. For example, one AFC participant described being at a point where they could no longer see a light at the end of the tunnel, and how the AFCSPS had changed this:

As I say to her, we took one thing off at a time, but for the first time, if you had spoken to me probably six months ago, three months ago, there was no light at this end of this [tunnel]… she genuinely has helped. (AFC3)

Yes, I’m feeling a bit more optimistic than I have been. I was getting to a point of total despair. (AFC4)

## Discussion

The aim of this first stage of evaluation was to understand the perspectives of AFC service users and the AFC SPLWs. This is the first time these experiences have been documented to understand how to effectively tailor social prescribing for the AFC.

The idea of the link worker representing the community they are serving is a fundamental aspect of a successful social prescribing provision.^39^ A key strength of the AFCSPS was that the AFC SPLWs were themselves veterans and could therefore draw on their own Armed Forces identity and experiences of transition from an Armed Forces to a civilian self. Both the service users and AFC SPLWs expressed specific reasons why this provided additional benefits which are discussed further below.

The service users all highlighted how they did not have to explain to the AFC SPLW about the culture and identity associated with the Armed Forces.^4 40^ This enabled the AFCSPS service users to be comfortable in their Armed Forces identity, using language and ‘banter’ they knew would be understood, even trusted. Importantly, the AFC SPLWs understood the challenges faced when transitioning to civilian life, such as working out a new identity, the change or loss of structure, and living with the trauma and impact of being deployed.^11 40 41^

Interestingly, both the service users and the AFC SPLWs spoke of how their military identity would always be part of them. The AFC link workers, however, identified how many service users were still ‘clinging onto’ their military identity, despite having been a civilian for many years. This is an important issue to recognise as a more prominent military identity in veterans has been shown to negatively influence the amount of support they engage with.^42 43^ Furthermore, from the perspective of social identity theory,^44^ the inability to adapt to the life transitions that bring about identity changes can result in diminished social support, belonging, purpose and well-being. The AFCSPS service users were all experiencing the impacts of an inability to adapt to their new civilian identity, as they were referred to the AFCSPS for support with access to food, heating and housing issues, financial issues alongside social isolation, physical issues and mental health needs.

To help the AFC service users transition successfully out of a more prominent military identity into a more prominent civilian one, the AFC SPLW appeared to act as a bridge between these two cultures. For the AFCSPS service users, where asking for support can be difficult for them to do, knowing the AFC SPLW was a veteran instantly changed the conversation and they opened up much more about their situation. This finding adds to previous research in the USA showing that veterans who felt that their counsellor shared their veteran identity reported statistically significantly higher positive counsellor behaviours and levels of satisfaction with the programme of support compared with veterans who did not hold this belief.^45^

Recognising that loss of structure is a key issue in the participants’ transition to civilian life, the AFC SPLWs created some structure around the multiple needs the AFC service users had. The AFC SPLWs often dealt with one thing at a time and ensured they got the job done. This ‘one issue at a time’ approach was highly appreciated, as it felt like an approach used in the military. The SPLWs were also able to liaise with services and organisations in a manner that was expected by civilians. This removed the burden for veterans to have to interact with lots of services when struggling to articulate their support needs in appropriate civilian language. This approach resonates with findings by Finegan *et al*,^36^ from social prescribing interventions to reduce depression and suicide in the AFC. Reducing and solving one situational stressor in a timely and effective manner had a meaningful bearing on the individual and re-energised and motivated them to address other issues.

As well as navigating civilian structures and processes, the AFC SPLWs were able to support the participants to connect into social groups. Not all the groups were for veterans specifically but enabled veterans to connect with other likeminded individuals doing something that brings them a sense of enjoyment or purpose. An additional explanation that may contribute to the positive experiences of the AFCSPS service users comes from the aspect of mattering.^46^ Mattering is “the feeling that others depend on us, are interested in us, are concerned with our fate, or experience us as ego-extension”.^47^ Taylor and Turner’s^46^ longitudinal analysis of mattering to others showed that as mattering increased, depressive symptoms decreased. This was separate to the effect of social support. While in the military service, everybody has a role and experiences the feelings that others depend on them and, therefore, is likely to experience high levels of mattering. Once into civilian life, the identity shift can be profound and most participants in this evaluation experienced social isolation and, potentially, a sense that no one depended on them anymore. In trying to transition into a civilian identity, they now depended on others yet often reported difficulties in getting services to respond to them, or felt they were on yet another waiting list, so they did not matter to others. We propose that the impact of the AFC SPLWs supporting them gave them a renewed sense of mattering, via someone mattering about them and them becoming part of a new community and mattering to others.

None of the AFC participants made any suggestions on how to improve the service despite being given the opportunity during their interview. The AFC SPLWs highlighted a couple of areas that they had addressed to improve service provision as they went along. One challenge that emerged was identifying people who were pretending to be Armed Forces veterans aka ‘Walter Mittys’ and, therefore, were not entitled to the support offered. This issue led to checks being carried out on every person referred to ensure they were a bona fide veteran or related to a veteran. This has added additional admin time into the service.

There was also a constant tension for the SPLWs between providing enough support and not over-supporting service users as this risked the service users wanting to use the SPLWs as a befriending service. Partly, the SPLWs recognised their own military influences in ‘getting a job done’, and ‘going above and beyond’ and used each other to improve and evolve their approaches to closing the cases when the veteran-specific support had been completed. This awareness helps to avoid burnout for the SPLWs, which is an issue documented in some social prescribing services, for example.^48^

Overall, this combination of military-identity inspired approaches to personalised support resulted in the AFCSPS service users feeling like there was hope again, and feeling more optimistic, as well as having improved social determinants of health, overall well-being and increased social interactions.

There are several limitations with this evaluation. Only five service users were interviewed, so the data here may not represent the views of all the AFCSPS service users. All were veterans; therefore, other members of the AFC, that is, spouses and dependents, were not represented in this interview sample. Due to the size of the service, we were also only able to interview two previously employed SPLWs and those currently delivering the service. Our findings will be influenced by the sampling methodology and difficulty in recruiting AFC individuals for research, especially where some are still in crisis or do not wish to disclose or talk about their own specific experiences. Further research should, therefore, be carried out to confirm these findings.

## Conclusion

The AFCSPS service is unique in its approach to supporting the AFC. Using SPLWs who come from the AFC provides a deep cultural understanding of how identity is associated with being in the Armed Forces. AFC services appreciated this level of understanding which enabled them to engage more fully with the support being offered. AFC SPLWs helped AFC service users with practical issues associated with transition from the Armed Forces to civilian life, moved beyond foundational aspects like trust and respect, to address those crucial elements associated with determinants of health, isolation and broader well-being. As a result, AFC had a renewed sense of hope and optimism, particularly for those AFC participants facing extreme hardship and/or experiencing despair. Ultimately, AFC participants reported improved psychological and physical well-being.

## Supplementary material

10.1136/bmjoq-2025-003842 online supplemental figure 1

10.1136/bmjoq-2025-003842 online supplemental figure 2

10.1136/bmjoq-2025-003842 online supplemental table 1

## Data Availability

No data are available.

## References

[1] Oster C, Morello A, Venning A (2017). The health and wellbeing needs of veterans: a rapid review. BMC Psychiatry.

[2] Rahnejat AM, Ebrahimi M, Khoshdel A (2022). The prevalence of depression among iran-iraq war veterans, combatants and former prisoners of war: A systematic review and meta-analysis. Int J Psychol.

[3] Randles R, Burroughs H, Green N (2025). Prevalence and risk factors of suicide and suicidal ideation in veterans who served in the British Armed Forces: a systematic review. BMJ Mil Health.

[4] McKenzie A, Burdett H, Croak B (2023). Adjustment disorder in the Armed Forces: a systematic review. *J Ment Health*.

[5] Smith RT, True G (2014). Warring Identities. Soc Ment Health.

[6] Brunger H, Serrato J, Ogden J (2013). “No man’s land”: the transition to civilian life. J Aggress Confl Peace Res.

[7] Morris J, Hanna P (2023). The Military to Civilian Transition: Exploring Experiences of Transitions to ‘Civvy Street’ and Implications for the Self. JVS.

[8] Ahern J, Worthen M, Masters J (2015). The Challenges of Afghanistan and Iraq Veterans’ Transition from Military to Civilian Life and Approaches to Reconnection. PLoS ONE.

[9] Randles R, Finnegan A (2022). Veteran help-seeking behaviour for mental health issues: a systematic review. BMJ Mil Health.

[10] Bergman BP, Mackay DF, Pell JP (2022). Suicide among Scottish military veterans: follow-up and trends. Occup Environ Med.

[11] Harden L, Murphy D (2018). Risk factors of suicidal ideation in a population of UK military veterans seeking support for mental health difficulties. J R Army Med Corps.

[12] Rodway C, Ibrahim S, Westhead J (2023). Suicide after leaving the UK Armed Forces 1996-2018: A cohort study. PLoS Med.

[13] Rozanov V, Carli V (2012). Suicide among war veterans. Int J Environ Res Public Health.

[14] Hitch C, Toner P, Armour C (2023). Enablers and barriers to military veterans seeking help for mental health and alcohol difficulties: A systematic review of the quantitative evidence. *J Health Serv Res Policy*.

[15] Ein N, Gervasio J, St. Cyr K (2024). A rapid review of the barriers and facilitators of mental health service access among Veterans and their families. Front Health Serv.

[16] NHS England (2019). The NHS long term plan.

[17] Sanderson J, Kay N, Watts R (2019). Universal personalised care: the implementing the comprehensive model.

[18] Polley M, Fixsen A, Seers H (2021). Evaluation of a Social Prescribing Pilot in Shropshire – implementation and impact findings. Eur J Integr Med.

[19] Polley M, Seers H, Johnson R (2021). Tandridge district council wellbeing prescription service final evaluation report.

[20] Muhl C, Mulligan K, Bayoumi I Establishing internationally accepted conceptual and operational definitions of social prescribing through expert consensus: a delphi study. Public and Global Health.

[21] Muhl C, Mulligan K, Giurca BC (2024). Building common understanding: seeking consensus and defining social prescribing across contexts - a collective commentary on a Delphi study. BMC Health Serv Res.

[22] Bertotti M, Frostick C, Temirov O (2020). An evaluation of social prescribing in the London borough of redbridge: final evaluation report.

[23] Dayson C, Damm C (2020). Evaluation of the rotherham social prescribing service for long term conditions.

[24] Wildman J, Wildman JM (2021). Evaluation of a Community Health Worker Social Prescribing Program Among UK Patients With Type 2 Diabetes. JAMA Netw Open.

[25] Wildman JM, Moffatt S, Steer M (2019). Service-users’ perspectives of link worker social prescribing: a qualitative follow-up study. BMC Public Health.

[26] Husk K, Blockley K, Lovell R (2020). What approaches to social prescribing work, for whom, and in what circumstances? A realist review.

[27] Cooper M, Avery L, Scott J (2022). Effectiveness and active ingredients of social prescribing interventions targeting mental health: a systematic review. BMJ Open.

[28] Bu F, Hayes D, Burton A (2024). Equal, equitable or exacerbating inequalities: patterns and predictors of social prescribing referrals in 160 128 UK patients. Br J Psychiatry.

[29] Cornwall, Council (2025). UK armed forces - veterans.

[30] Jarvis L (2022). Armed forces community social prescribing demonstrator end of year report 2022.

[31] Wakefield JRH, Kellezi B, Stevenson C (2022). Social Prescribing as “Social Cure”: A longitudinal study of the health benefits of social connectedness within a Social Prescribing pathway. J Health Psychol.

[32] Polley MJ, Seers H, Toye O (2023). Building the economic case for social prescribing.

[33] Costa A, Sousa CJ, Seabra PRC (2021). Effectiveness of social prescribing programs in the primary health-care context: a systematic literature review.

[34] Chatterjee HJ, Camic PM, Lockyer B (2018). Non-clinical community interventions: a systematised review of social prescribing schemes.

[35] Kimberlee R (2015). What is social prescribing?. *ASSRJ*.

[36] Finnegan A, Salem K, Ainsworth-Moore L (2025). “One Is Too Many” preventing self-harm and suicide in military veterans: a quantitative evaluation. BMJ Mil Health.

[37] Hazeldine E, Gowan G, Wigglesworth R (2021). Link worker perspectives of early implementation of social prescribing: A “Researcher-in-Residence” study. Health Soc Care Community.

[38] Clarke V, Braun V (2017). Thematic analysis. J Posit Psychol.

[39] Polley M, Dixon M, Pilkington K (2016). Report of the annual national social prescribing network conference.

[40] Hall LK (2011). The importance of understanding military culture. Soc Work Health Care.

[41] Demers A (2011). When Veterans Return: The Role of Community in Reintegration. J Loss Trauma.

[42] Gorman JA, Scoglio AAJ, Smolinsky J (2018). Veteran Coffee Socials: A Community-Building Strategy for Enhancing Community Reintegration of Veterans. Community Ment Health J.

[43] Harada ND, Villa VM, Reifel N (2005). Exploring veteran identity and health services use among Native American veterans. Mil Med.

[44] Haslam C, Haslam SA, Jetten J (2021). Life Change, Social Identity, and Health. Annu Rev Psychol.

[45] Gade DM, Wilkins VM (2013). Where Did You Serve? Veteran Identity, Representative Bureaucracy, and Vocational Rehabilitation. J Public Adm Res Theory.

[46] Taylor J, Turner RJ (2001). A longitudinal study of the role and significance of mattering to others for depressive symptoms. J Health Soc Behav.

[47] Rosenberg M, McCullough B (1981). Mattering: Inferred Significance and Mental Health among Adolescents. Res Community Ment Health.

[48] Rhodes J, Bell S (2021). “‘It sounded a lot simpler on the job description’”: A qualitative study exploring the role of social prescribing link workers and their training and support needs (2020). Health Soc Care Community.

[49] Dixon N (2017). Guide to managing ethical issues in quality improvement or clinical audit projects.

