# Supplementary material for: Qualitative exploration of service users and social prescribing link workers of the Armed Forces Community social prescribing scheme in Cornwall
Source: BMJ Open Qual. 2026 Feb 3;15(1):e003842. doi: 10.1136/bmjoq-2025-003842 (PMC12878429; doi:10.1136/bmjoq-2025-003842)
Supplement: online supplemental figure 1 [file bmjoq-15-1-s001.pdf]

TIDieR

Quality study of a armed forces community (AFC) social prescribing link worker (SPLW) service

**Why:**	The armed forces community experience significant health inequalities and a range of barriers to accessing services for their health and wellbeing when compared to the general population. For this reason, this intervention provided:<br><br>· Advocacy, connector and navigation services to members of the armed forces community.<br>· Training, education and mentoring services for social prescribers and allied health professionals and wider VCSE.<br>· Raise community awareness and increase the number of Veteran Friendly accredited organisations.
**What (material):**	The social prescribing services is delivered by two SPLWs who have lived experience of serving in the armed forces. To support wider needs of this population, the SPLWs were trained in for examples: mental Health and suicide prevention; substance misuse awareness; domestic abuse and sexual violence awareness; Identification and Brief Advice (IBA) to reduce alcohol consumption and smoking; Making Every Contact Count (MECC); coaching and safeguarding.
**What (procedures):**	SPLWs worked with individuals to co-produce a personalised action plan that was tailored to each person with an aim to improve their health and wellbeing outcomes. Goal setting was used as part of the plan to help support people, as well as provide relevant signposting to other services and support that is available.
**Who provided:**	Using lived experience and knowledge of the support available to the armed forces community, the two AFC SPLWs worked with individuals to connect and support them accessing a range of statutory and community support. These could include accessing support available for both physical and mental health needs, finance/debt, housing, lifestyle factors such as drug and alcohol treatment, domestic abuse and sexual violence.
**How (mode of delivery; individual or group):**	The SPLW intervention consisted of an initial face to face or virtual consultation where the social prescribers worked with individuals for between 1 – 1.5 hours to understand their individual needs and to co-produce a personalised care plan. Subsequent meetings were offered for up to 12 months or when there was no further use for social prescribing with follow-up appointment lasting up to 1 hour. Due to the complexity of the health and wellbeing needs of this population, there were no restrictions as long as individuals could benefit from social prescribing. Where individuals presented in crisis or with needs outside the scope of social prescribing, the SPLWs worked with them to access relevant services such as those associated with mental health, drug and alcohol treatment.
**Where:**	Cornwall has one of the largest veteran populations in the UK. For this reason, the two SPLWs worked across both Cornwall and the Isles of Scilly.
**When and how much:**	Due to the variable needs of the armed forces community population, there were no restrictions on the number of appointments as described above. Also, this depended on the types of support individuals were sign posted to.
**Tailoring:**	Personalised care is central to this intervention so that the offer and action plan was tailored to each individual.
**Modification:**	

This is a prevention programme to support the armed forces community who can experience a range of multiple vulnerabilities, which means some individuals can reach crisis and are referred into the service. The intervention is not designed to support those in crisis. When this happens there is an escalation and safeguarding process in place to ensure individuals are supported by the right organisation.

How well (planned): All referrals into the service were screened for their appropriateness for a social prescribing intervention. Then all individuals were followed up by the two SPLWs.

How well (actual): The AFC SPLW intervention was delivered as intended. Result outcomes of the intervention was dependent and could be affected by the individuals accessing the service. This may be because of higher levels of support needs outside the scope of the intervention (e.g., mental health crisis, drug and alcohol treatment), previous trauma that may act as a barrier to reaching out for support (reduced by the SPLWs having lived experience) and natural drop out of those not wishing to engage.
