# Supplementary material for: Qualitative exploration of service users and social prescribing link workers of the Armed Forces Community social prescribing scheme in Cornwall
Source: BMJ Open Qual. 2026 Feb 3;15(1):e003842. doi: 10.1136/bmjoq-2025-003842 (PMC12878429; doi:10.1136/bmjoq-2025-003842)
Supplement: online supplemental figure 2 [file bmjoq-15-1-s002.pdf]

Meaningful Measures

Interview Schedule Armed Forces Community Social Prescribing service users

Openers
Remind me, which force did you serve with, what was your role, when did you leave?

When did you first start to use the social prescribing service?

Key questions:

1. Do you feel you have benefited as a result of using the Armed Forces Community Social Prescribing Service?

   Follow up Perhaps you can tell me a bit about the different ways you have benefitted?

2. **This has been quite a new service and we are keen to understand what has been most important to you about the service?**

   Follow up question - is there any particular reason why / can you tell me a bit more about…

3. **Have you seen other benefits or ripple effects as a result of the initial support received?**

4. **If you had a magic wand, is there anything about the service that you would change or develop?**

Interview Schedule Armed Forces Community Social Prescribing Link workers

Their background:

Can you tell me a little about your professional background?

- What qualifications, or areas of work?
    - How did you get into social prescribing?
    - What was your motivation for doing it?

Their role as an SPLW within the Armed Forces Community Social Prescribing service?

- How long have you been associated with the AFCSPS?
- What does that involve/entail

Service users:

What types of service users do you see?

- Any typical demographics?

How are people referred to your service?

- Does this way of referral work?
- Are there any routes you would like to develop?

What type of problems and issues are you most commonly supporting when you meet with service users?

4. Are there any challenges associated with this?

When you talk to a service user, do they usually need support for the reason they were referred to you for, or does the priority change as the conversation unfolds?

What about the family members of veterans; do you get any of these using the service?
If yes,

5. How are they referred?
6. What are the most common reasons they come to you for?
7. Any challenges?

Benefits of the service:

In your experience what have been the positive impacts of the social prescribing service for the service users?

Any challenges (e.g., help-seeking, stigma, lack of knowledge, misunderstanding of SP, limited resources available)?

Service development:

Thinking about the social prescribing service as a whole, are there any areas or aspects that you want to change or improve?

Also any areas that are working really well?

Anything you want to add that I haven't asked?

.
