# Supplementary material for: Qualitative exploration of service users and social prescribing link workers of the Armed Forces Community social prescribing scheme in Cornwall
Source: BMJ Open Qual. 2026 Feb 3;15(1):e003842. doi: 10.1136/bmjoq-2025-003842 (PMC12878429; doi:10.1136/bmjoq-2025-003842)
Supplement: online supplemental table 1 [file bmjoq-15-1-s003.pdf]

Category	Armed Forces Community Social Prescribing Service	Civilian Social Prescribing Service
**Coverage**	Cornwall and Isles of Scilly are covered by 2 social prescribing link workers. This is because the armed forces community are a specific minority of the population	Usually civilian SPLWs work within a much smaller geography ie 1-2 primary care networks. This is because most people in the locality will be eligible for social prescribing support unless the scheme has specific eligibility criteria.
**Remit**	To address issues that are specifically related to serving in the armed forces for either a Veteran or their family members.	Supporting all types of concerns and issues for the general public. Social prescribing link workers can refer into AFC SPS for additional support for the armed forces community.
**Approach**	AFC SPLW's uses a whole family approach. This means that they are able to support the Veteran as well as the wider family unit, including but not limited to spouses, children, and widows of Veterans.<br><br>When a Veteran is experiencing challenges, it is often the case that their family members are also affected and may require support themselves. The AFC SPLW can offer combined family focused support, this ensures that everyone's needs are recognised and addressed.	Usually supporting one person and not necessarily additional family members
**Awareness building**	Part of the AFC social prescribing link worker role is to build awareness of the armed forces community needs within Cornwall Isles of Scilly and to increase the number of veteran friendly accredited organisations.<br>The AFCS SPLW's have played a key role in helping Cornwall and the Isles of Scilly achieve 100% Veteran Accreditation across all GP surgeries.	Where possible link workers attend community events to raise awareness of the social prescribing service and set up new relationships with organisations.

**Where are the social prescribing link workers employed?**	AFC social prescribing link workers are employed by Active Plus CIC, with funding coming from Cornwall Council.	The majority of social prescribing schemes are in the NHS via Primary Care Networks. Much less are provided by the local authority or housed in the Voluntary, Community Faith and Social Enterprise sector.
**Who are the link workers**	AFC social prescribing link workers are themselves armed forces veterans. Additionally, Both AFC SPLW 's are married to veterans and bring a wealth of lived experience to the role. They understand the realities of military life, its pressures, and the impact it has on families, which enables them to engage with clients sensitively, helping them to build meaningful rapport, and provide informed and compassionate support.	Civilian social prescribing link workers do not usually have any armed forces experience or have Link lived experience of serving or being the partner of someone who has been in active service.

Supplementary Table 1. Comparison between the Armed Forces Community and Civilian Social Prescribing Schemes.
